# Endoscopic Management of Post-Esophagectomy Delayed Gastric Conduit Emptying (DGCE): Results from a Cohort Study in a Tertiary Referral Center with Comparison between Procedures

**DOI:** 10.3390/cancers16203457

**Published:** 2024-10-12

**Authors:** Giuseppe Dell’Anna, Francesco Vito Mandarino, Jacopo Fanizza, Ernesto Fasulo, Alberto Barchi, Rukaia Barà, Edoardo Vespa, Edi Viale, Francesco Azzolini, Lorella Fanti, Silvia Battaglia, Francesco Puccetti, Andrea Cossu, Ugo Elmore, Lorenzo Fuccio, Vito Annese, Alberto Malesci, Riccardo Rosati, Silvio Danese

**Affiliations:** 1 Gastroenterology and Gastrointestinal Endoscopy Unit, IRCCS San Raffaele Hospital, Via Olgettina 60, 20132 Milan, Italy; mandarino.francesco@hsr.it (F.V.M.); fanizza.jacopo@hsr.it (J.F.); fasulo.ernesto@hsr.it (E.F.); barchi.alberto@hsr.it (A.B.); bara.rukaia@hsr.it (R.B.); vespa.edoardo@hsr.it (E.V.); viale.edi@hsr.it (E.V.); azzolini.francesco@hsr.it (F.A.); fanti.lorella@hsr.it (L.F.); malesci.alberto@hsr.it (A.M.); danese.silvio@hsr.it (S.D.); 2 Gastroenterology and Gastrointestinal Endoscopy Unit, IRCCS Policlinico San Donato, Piazza Edmondo Malan 2, 20097 San Donato Milanese, Italy; vito.annese@grupposandonato.it; 3 Faculty of Medicine and Surgery, Vita-Salute San Raffaele University, Via Olgettina 56, 20132 Milan, Italy; elmore.ugo@hsr.it (U.E.); rosati.riccardo@hsr.it (R.R.); 4 Gastrointestinal Surgery Unit, IRCCS San Raffaele Hospital, Via Olgettina 60, 20132 Milan, Italy; battaglia.silvia@hsr.it (S.B.); puccetti.francesco@hsr.it (F.P.); cossu.andrea@hsr.it (A.C.); 5 Unit of Gastroenterology, Department of Medical and Surgical Sciences, S. Orsola-Malpighi University Hospital, University of Bologna, Via Massarenti 9, 40138 Bologna, Italy; lorenzofuccio@gmail.com

**Keywords:** delayed gastric conduit emptying, esophagectomy, endoscopic pneumatic dilation, botulinum toxin

## Abstract

**Simple Summary:**

Delayed gastric conduit emptying (DGCE) is a common complication of esophageal surgery. The current study compares three endoscopic procedures—Intra-Pyloric Injection of Botulinum Toxin (IPBT), Pneumatic Balloon Dilation (PBD), and a combination of both in the same session (BTPD)—to determine which approach is the most effective in DGCE treatment. By analyzing data from 64 patients endoscopically treated, results showed that the combination approach (BTPD) was associated with a higher rate of symptom resolution. BTPD allowed patients to resume eating and be discharged more quickly. These findings suggest that BTPD may be the most effective treatment for DGCE, offering better patient outcomes and potentially guiding future treatment strategies.

**Abstract:**

**Background/Objectives**: Delayed gastric conduit emptying (DGCE) occurs in 15–39% of patients who undergo esophagectomy. Intra-Pyloric Injection of Botulinum Toxin (IPBT), Pneumatic Balloon Dilation (PBD), and the same session combination (BTPD) represent the main endoscopic procedures, but comparative data are currently unavailable. **Methods**: We retrospectively analyzed prospectively collected data on all consecutive patients with DGCE treated endoscopically with IPBT, PBD, or BTPD. ISDE Diagnostic Criteria were used for DGCE diagnosis and classification. A Gastric Outlet Obstruction Score was used for clinical staging. All patients undergoing IPBT received 100 UI of toxin, while those undergoing PBD were dilated up to 20 mm. Clinical success (CS) was defined as the resolution of symptoms/resumption of feeding at discharge or expanding dietary intake at any rate. Recurrence was defined as symptom relapse after more than 15 days of well-being requiring endoscopic/surgical intervention. **Results**: A total of 64 patients (81.2% male, 90.6% Ivor-Lewis esophagectomy, 77.4% adenocarcinoma) with a median age of 62 years (IQR 55–70) were enrolled: 18 (28.1%) in the IPBT group, 24 (37.5%) in the PBD group, and 22 (34.4%) in the BTPD group. No statistically significant differences were found in the baseline characteristics, surgical techniques, and median follow-up among the three groups. BTPD showed a higher CS rate (100%) compared to the PD and BTPD groups (*p* = 0.02), and a Kaplan–Meier analysis with a log–rank test revealed that the BTPD group was associated both with a significatively shorter mean time to refeed of 1.16 days (95% CI 0.8–1.5; *p* = 0.001) and a shorter median time to discharge of one day (95% CI 1–3; *p* = 0.0001). **Conclusions**: Endoscopic management of DGCE remains challenging. Waiting for further strong evidence, BTPD can offer patients a higher clinical efficacy rate and a shorter time to refeed and be discharged.

## 1. Introduction

In the context of multimodal treatment for esophageal carcinoma, surgical resection currently represents the only curative intent therapy [1]. Specifically, in cases of distal esophageal neoplasms, Ivor-Lewis Esophagectomy (IL-E) with gastric conduit reconstruction is the preferred surgical procedure [2]. However, this surgery is burdened with a series of short- and long-term complications with significant morbidity, including anastomotic leaks (AL), conduit necrosis, anastomotic strictures, and delayed gastric conduit emptying (DGCE) [3,4,5,6]. The pathophysiology of DGCE remains unclear, although increasing evidence demonstrates a multifactorial etiology involving multiple factors. The primary factors include dysfunctions in peristalsis and pyloric release (vagotomy), an unfavorable pressure gradient (negative pressure in the chest and positive pressure in the abdomen), conduit angulation that may also be redundant, reduced esophageal hiatus width, and altered gastric microbiota and function/release of GUT hormones [4,7,8].

The limitations of the current literature on DGCE have primarily been associated with the absence of shared diagnostic criteria and symptom grading tools, which the International Society for Diseases of the Esophagus (ISDE) recently proposed and published, identifying an early form (E-DGCE) and a late form (L-DGCE), aiming to overcome these limitations (Table 1) [9].

To further standardize the management of DGCE, a diagnostic protocol utilizing an Upper Gastrointestinal (UGI) contrast study has recently been advocated and incorporated within the dedicated ERAS protocol [10].

DGCE has an incidence ranging from 10 to 50% and can lead to short-term complications (AL and aspiration pneumonia) or long-term complications (malnutrition) that impact the patient’s quality of life [7,11]. Current evidence has not demonstrated a clear clinical benefit derived from prophylactic intraoperative pyloric drainage (IPD). On the contrary, such procedures, including finger fracturing, pyloroplasty, and pyloromyotomy, have been highlighted to be associated, in some cases, with complications such as duodenal leaks, dumping syndrome, and bile reflux [12]. More robust data on this topic will likely come from ongoing randomized controlled trials (RCTs) [12]. Since prokinetic drugs in this setting have proven to be ineffective, endoscopy thus plays an important role. The Intra-Pyloric Injection of Botulinum Toxin (IPBT), through its inhibitory effect on gastric and pyloric smooth muscles, is moderately effective in the short-term postoperative period. However, in the long term, it is characterized by a considerable recurrence rate of symptoms requiring endoscopic or surgical interventions [13,14]. Pneumatic balloon dilation of the pylorus (PBD) represents a viable alternative both in the short-term postoperative and long-term [15]. Recently, the possibility of simultaneously performing IPBT and PBD (BTPD) has been proposed to optimize the myorelaxant effect with the mechanical effect of dilation [16]. The currently available evidence presents several limitations regarding patient selection, the definition of clinical outcomes, and the lack of comparative data. Based on data from our tertiary care center, our study aims to show comparative data on the efficacy and safety of the endoscopic treatments currently most widely used in managing DGCE after esophagectomy.

## 2. Materials and Methods

### 2.1. Study Population and Ethical Approval

This retrospective single-center cohort study was conducted at the Gastroenterology and Gastrointestinal Endoscopy Division of IRCCS San Raffaele Hospital and University in Milan, following the Declaration of Helsinki (as revised in 2013). We performed a retrospective analysis of prospectively collected data into a prospective registry active at our center, approved by the local Ethical Committee (Protocol ID: REG_EGDS COLON; Approval Code: 79/12/2022), including all data about diagnostic and operative luminal endoscopic procedures. We recorded demographic, clinicopathological, surgical, endoscopic, and follow-up data in an electronic endoscopic database and patient hospital electronic medical records.

### 2.2. Patients

To analyze and compare the efficacy and safety of the three endoscopic procedures for DGCE management (IPBT, PBD, and BTBD), we enrolled all consecutive adult patients (>18 years old) affected by DGCE refractory to prokinetic drugs that were referred to our Endoscopy Unit from the Gastrointestinal Surgery Unit of our University Hospital (IRCCS San Raffaele Hospital, Milan, Italy), which underwent endoscopic DGCE management, from January 2014 to November 2023. For the DGCE diagnosis and classification, we referred to recently published DGCE diagnostic criteria by Konradsson et al. in 2020 (Table 1) [9]. For patients treated before the publication of these diagnostic criteria, we applied them retrospectively and included only patients with a confirmed DGCE diagnosis.

To assess the quality and consistency of feeding tolerated by patients, we used the Gastric Outlet Obstruction Score (GOOS), which gives a point of which provides a score from 0 to 3, depending on whether the patient does not eat (0), eats only a liquid diet (1), soft diet (2), or free diet (3) [17,18]. According to oncological and surgical indications, the GOOS was collected at diagnosis, discharge, and the last clinical follow-up.

### 2.3. Surgical Interventions

All surgical interventions were performed by the same surgical team experienced in esophagogastric and thoracic surgery. All surgical data were collected, including types of surgery (Ivor-Lewis Esophagectomy, McKeown Esophagectomy, total esophagectomy), the approach (Open, Laparoscopic, Hybrid), the site of the esophagogastric anastomosis (cervical, thoracic), IPD (finger fracture, pyloroplasty, pyloromyotomy) and eventually other surgery-related complications (e.g., AL, anastomotic stricture, conduit ischemia/necrosis).

### 2.4. Endoscopic Procedures

All endoscopic procedures were performed by expert endoscopists under deep sedation with anesthesiologic assistance, with the patient positioned supine on the left side in an operative endoscopy room equipped with fluoroscopy. Endoscopic procedural choice was mainly linked to endoscopist preference, always shared with surgeons after multidisciplinary discussion. If the patient had a nasogastric tube (NGT), it was removed immediately before starting the endoscopic procedure. A therapeutic gastroscope was used to provide a larger operative channel for the potential aspiration of food residues and secretions. Before the therapeutic endoscopic maneuver, a diagnostic esophagogastroduodenoscopy (EGD) was conducted to rule out other complications. IPBT was performed using a 25-gauge endoscopic injection needle. A vial of 100 units (U) of BT was mixed with 4 cc of normal saline, and 25 U of BT/mL was injected into the four quadrants of the pylorus. PBD was always performed with the same through-the-scope pneumatic balloon (Boston Scientific, CRE PRO Wireguided). Before dilation, a guidewire preloaded in the balloon catheter was advanced into the duodenum. Once the balloon was positioned across the pylorus, it was progressively inflated to 18, 19, and 20 mm, with the balloon being kept inflated for 60 s at each size. All these procedures were performed under endoscopic control, and fluoroscopy was never necessary. A final endoscopic check was always conducted to rule out any possible adverse events (AEs). In the combined procedure (BTPD), IPBT was performed first, followed by PBD.

### 2.5. Definitions

Technical success (TS) was defined as the completion of the scheduled endoscopic procedure. On the other hand, clinical success (CS) was defined as the resolution of DGCE symptoms (those included in the recently published diagnostic criteria) with the resumption of feeding (at least GOOS 2, soft solid) at discharge OR expanding dietary intake at any rate (always taking as reference GOOS score), without the need to replace the NGT [9]. For patients who achieved CS, recurrence was defined as relapse of DGCE symptoms after more than 15 days of well-being requiring intervention (nasogastric tube/endoscopic procedure/surgery). Endoscopic procedure-related AEs were classified according to the recent classification for Adverse events Gastrointestinal Endoscopy (AGREE) [19].

### 2.6. Statistical Analysis

All the statistical analyses were performed by MedCalc Version 18 (MedCalc Software, Ostend, Belgium). The normality of continuous variable distribution was tested with the Shapiro–Wilk test. Mean ± standard deviation (SD) and median and interquartile range (IQR) were used to express normally and non-normally distributed variables, respectively. Categorical parameters were compared using Pearson Chi-square test and Fisher exact test, along with Bonferroni correction for two or more groups within a variable, while continuous variables were compared using Pearson or Spearman correlation according to normality distribution, while by Student t-test or ANOVA test between two or more groups for normally distributed data, Mann–Whitney test or Kruskal–Wallis test between two or more groups, for non-normally distributed data. All reported *p* values were two-sided, with statistical significance set at 0.05. Time to refeed and time to discharge were analyzed by using the Kaplan–Meier method, in which patients were censored at recurrence, death, and last follow-up visit, whichever came first. A comparison of time-to-event curves was performed using the log–rank test.

## 3. Results

### 3.1. Baseline Characteristics

During the study period, 64 patients affected by DGCE were referred to our endoscopy unit and enrolled. Most patients are male (81.2%, 52/64), with a median age of 62 years (IQR 55–70). Regarding comorbidities at the time of esophageal surgery, most patients presented an American Society of Anesthesiologists score (ASA) of two (54.7%, 35/64). The most common indication for surgery was a malignant reason (96.9%, 62/64), and esophageal adenocarcinoma (EAC) represented the most common one (77.4%, 48/62), followed by squamous cell carcinoma (21.0%, 13/62) and leiomyoma (1.6%, 1/62). Concerning benign indications, both cases underwent esophageal surgery for perforations that were not amenable endoscopically. According to oncological staging at the diagnosis, most of the patients were classified as locally advanced (75.8%, 47/62). A total of 47 (75.8%) patients underwent neoadjuvant treatments at diagnosis: 40.3% (25/62) chemotherapy (CT), 33.9% (21/62) chemo-radiotherapy (CRT), and one patient (1.6%) radiotherapy (RT). Ivor-Lewis Esophagectomy (ILE) was the most performed surgical intervention (90.6%, 58/64), followed by McKeown Esophagectomy (MKE) (4.7%, 3/64) and total esophagectomy (4.7%, 3/64). In 87.5% (56/64) of cases, a minimally invasive approach (laparoscopic + thoracoscopic) was selected to perform surgery. According to the recent ISDE classification, almost two-thirds (67.2%, 43/64) of the patients included in the study were suffering from late DGCE (L-DGCE) [9]. Baseline characteristics of the entire study population are resumed in Table 2.

### 3.2. Comparison

In the entire cohort, 24 patients (37.5%) underwent PBD, 22 patients (34.4%) underwent BTPD, and 18 patients (28.1%) underwent IPBT. There were no significant differences between the groups in terms of sex (*p* = 0.61) or median age (*p* = 0.08). The three groups also appeared homogeneous regarding ASA score (*p* = 0.16) and comorbidities, particularly diabetes (*p* = 0.68), a known cause of gastroparesis [20]. Additionally, no differences were found between the groups concerning surgical indications (malignant vs. benign cause; *p* = 0.53), histology (esophageal adenocarcinoma, squamous cell carcinoma, leiomyoma; *p* = 0.58), staging (resectable, locally advanced, metastatic; *p* = 0.39), and neoadjuvant treatments (CT, RT, CRT; *p* = 0.17). A minimally invasive surgical approach was the most common in all three groups (88.8% in the IPTB group, 83.3% in the PBD group, and 90.9% in the BTPD group), with no statistically significant differences among the groups (*p* = 0.78). An important surgical aspect concerns IPD, where no significant differences were found between the treatment groups (*p* = 0.10). The combination of same-session pyloroplasty and pyloromyotomy was most common in the IPTB group (55.5%, 10/18) and the BTPD group (45.5%, 10/22). Regarding other surgical complications, such as AL, gastric conduit necrosis, or cardiopulmonary complications, no differences were observed between the groups (*p* = 0.12). Most patients in all three endoscopic groups did not experience complications, apart from DGCE (88.8% in the IPTB group, 66.7% in the PBD group, and 54.5% in the BTPD group). When comparing the timing of DGCE onset among the three groups, the rate of early DGCE was significantly higher in the IPTB group compared to the other two treatment groups (77.7% vs. 20.83% vs. 9.09%; *p* < 0.0001). In contrast, no differences were found between the groups when comparing the GOOS score before endoscopic treatment (*p* = 0.33). In both the IPTB (44.4%, 8/18) and PBD (50.0%, 12/24) groups, most patients had a GOOS score of one (liquid diet) before endoscopy. Conversely, in the BTPD group, many patients (36.36%, 8/22) had a GOOS score of two (soft solid diet). The results of the baseline characteristic comparisons between the three groups are reported in Table 3.

### 3.3. Outcomes

Despite a 100% TS rate across all three groups, the BTPD group demonstrated a significantly higher CS rate compared to the PBD and IPTB groups (100.0% BTPD vs. 91.6% PBD vs. 72.22% IPTB; *p* = 0.01). No significant differences were observed in the median follow-up times (days) among the groups (*p* = 0.19), with follow-ups of 374 days (IQR 208–739) for the IPTB group, 230 days (IQR 144–589) for the BTPD group, and 184 days (IQR 35–710) for the PBD group. Among patients who achieved CS after endoscopic treatment, the IPTB group had the highest rate of symptom recurrence compared to the other two groups, though this difference was not statistically significant [23.08% (3/13) in the IPTB group, 22.72% (5/22) in the PBD group, 9.09% (2/22) in the BTPD group; *p* = 0.4126]. Regarding rescue management, the three recurrent cases in the IPTB group were treated with Gastric-Peroral Endoscopic Myotomy (G-POEM), BTPD, and surgical pyloromyotomy with pyloroplasty, respectively. The five recurrent cases in the PBD group were managed with two BTPD, two PBD, and one IPTB. Finally, the two recurrent cases in the BTPD group were treated with G-POEM and surgical pyloromyotomy with pyloroplasty, respectively. No AEs related to endoscopic procedures were recorded during the study period.

**Figure 1:**
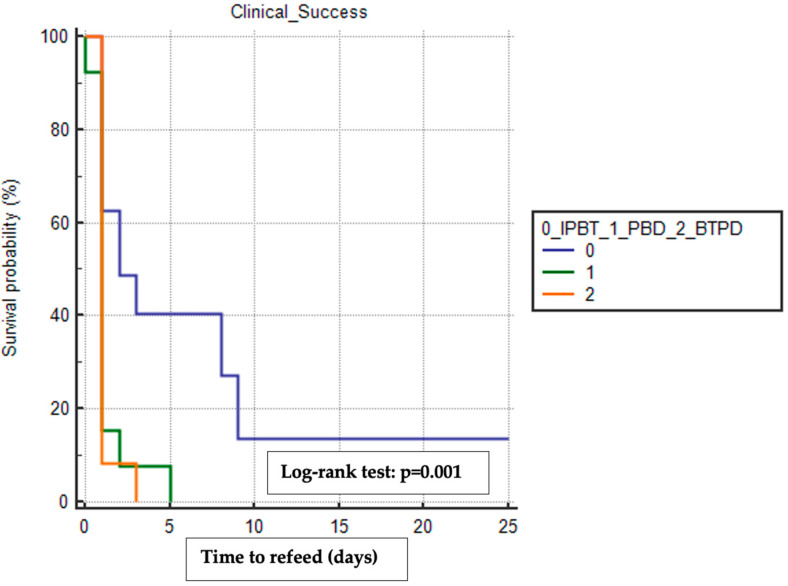
Kaplan–Meier analysis with the log–rank test of the difference in time to refeed between the three treatment groups. In this analysis were included only patients who reached clinical success and who did not experience other surgical complications. IPBT, intra-pyloric botulinum toxin injection. PBD, pneumatic balloon dilation. BTPD, Botulinum Toxin Pneumatic Dilation. The copyright of the image belongs to the authors.

We used the Kaplan–Meier method to estimate the mean time to refeeding and discharge among the treatment groups, including only patients who achieved clinical success and excluding those who experienced other surgical complications (e.g., anastomotic leak, conduit necrosis, pulmonary disorders, anastomotic stricture). The BTPD group had the shortest mean time to refeeding (1.17 days, 95% CI 0.84–1.49) compared to the PBD group (1.31 days, 95% CI 0.66–1.95) and the IPTB group (6.56 days, 95% CI 1.79–11.33), with a statistically significant difference observed when comparing the time-to-event curves using the log–rank test (*p* = 0.001) (Figure 1).

**Figure 2:**
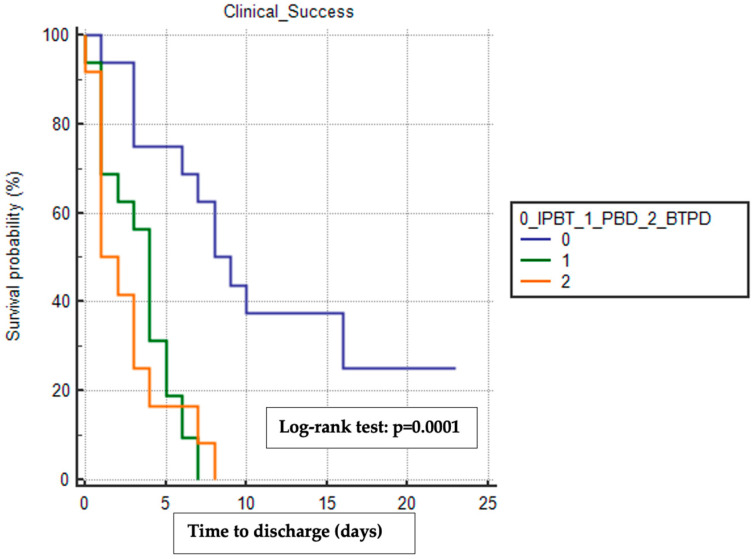
Kaplan–Meier analysis with the log–rank test of the difference in time to discharge between the three treatment groups. This analysis included only patients who reached clinical success and did not experience other surgical complications. IPBT, intra-pyloric botulinum toxin injection. PBD, pneumatic balloon dilation. BTPD, Botulinum Toxin Pneumatic Dilation. The copyright of the image belongs to the authors.

Similarly, the BTPD group had the shortest median time to discharge (1 day, 95% CI 1–3) compared to the PBD group (4 days, 95% CI 1–5) and the IPTB group (8 days, 95% CI 6–16), with a statistically significant difference observed (log–rank test *p* = 0.0001) (Figure 2).

The results of outcomes comparison between the three groups are reported in Table 4.

### 3.4. Subgroup Analysis

We also performed a subgroup analysis to reduce the risk of bias by comparing baseline characteristics and outcomes of patients treated with IPTB (n = 12) or BTPD (n = 14), specifically including only those who underwent ILE with IPD. The two subgroups did not differ in baseline characteristics (comorbidities, indication for surgery, neoadjuvant treatments, oncological staging), surgical aspects (surgical approach, types of IPD, surgical complications), or the timing of DGCE onset and subsequent disease classification (Table 5).

Data from the subgroup analysis further indicated that the BTPD subgroup had a significantly higher CS rate compared to the IPTB subgroup, with rates of 100% vs. 75%, respectively (*p* = 0.047). There was no difference in median follow-up between the two subgroups [313 days (IQR 208–608) for the IPTB subgroup vs. 197 days (IQR 65–499) for the BTPD subgroup; *p* = 0.12]. The IPTB subgroup also had a higher rate of symptom relapse compared to the BTPD subgroup (33.3% vs. 7.1%; *p* = 0.11) (Table 6).

**Figure 3:**
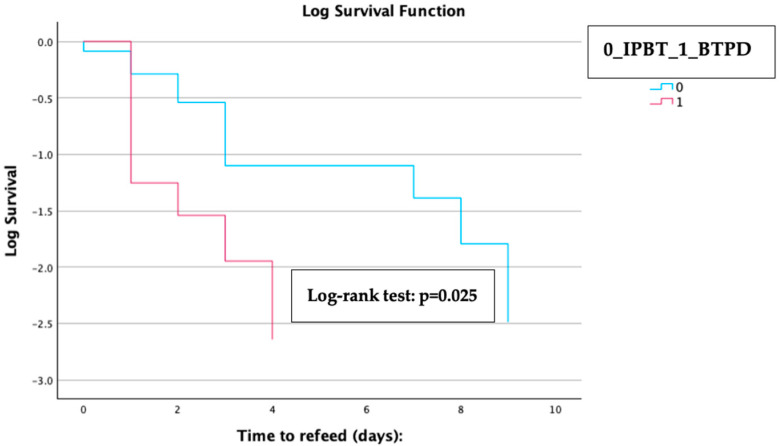
Kaplan–Meier analysis with the log–rank test of the difference in time to refeed between the two treatment groups included in the subgroup analysis. The copyright of the image belongs to the authors.

Kaplan–Meier analysis with a log–rank test revealed that patients in the BTPD subgroup experienced a significantly shorter median time to refeeding compared to those in the IPTB subgroup [1 day (IQR 0.3–1.2) for the BTPD subgroup vs. 3 days (IQR 1.9–4.1) for the IPTB subgroup; log–rank test *p* = 0.025] (Figure 3).

## 4. Discussion

DGCE. is a common complication following esophagectomy, significantly impacting both short- and long-term outcomes. Various surgical and endoscopic IPD strategies have been attempted to prevent DGCE, but the results have been inconsistent [4,21]. Hajibandeh S et al., in their recent meta-analysis, found that intraoperative IPBT did not reduce the risk of DGCE or the need for endoscopic pyloric interventions compared to no intraoperative IPBT or pyloroplasty [22]. Similarly, Arya S et al., in a systematic review, did not find any difference in DGCE risk between patients who underwent various types of IPD (e.g., IPBT, pyloroplasty, pyloromyotomy, finger fracture) and those who did not [23]. Conversely, Loo JH et al., in their meta-analysis, demonstrated that IPD, including pyloroplasty, pyloromyotomy, and IPBT, was associated with a reduced risk of developing DGCE [24]. Supporting this, a meta-analysis by Abdelrahman M et al. showed that intraoperative PBD significantly reduces the rate of early DGCE and AL following esophagectomy [25].

As with other complications of esophagogastric surgery, endoscopy plays a key role in this setting [26]. The evidence for the endoscopic treatment of DGCE is currently limited to small retrospective series that lack standardization in inclusion criteria and treatment protocols. For example, Mertens A et al. evaluated PBD in managing early DGCE (<14 postoperative days) in a retrospective single-center series of 12 patients, reporting a CS rate of 58% [15]. Bhutani MS et al. recently published results from a series of 21 patients on the efficacy and safety of same-session combination BTPD in treating DGCE, showing a clinical efficacy rate of 85% [16]. G-POEM has also shown promise in this setting, but the evidence is limited to case reports and small case series [27,28,29,30].

These studies are primarily limited by a lack of standardization in the definition and evaluation of DGCE, outcomes assessment, and the absence of comparative data. Diagnostic criteria for DGCE were recently published by ISDE to assist clinicians in better diagnosing and classifying this complication and to provide researchers with a valuable tool for standardizing their results. In this context, our single-center cohort study aimed to evaluate the safety and efficacy of the most common endoscopic procedures for treating DGCE: IPBT, PBD, and same-session BTPD. For the first time, our study applied the ISDE Diagnostic Criteria for DGCE diagnosis and classification to minimize selection bias among included patients. Our results demonstrated that the BTPD group not only had significantly higher clinical efficacy but also experienced shorter times to refeeding and hospital discharge, with no differences in safety outcomes. We also performed a subgroup analysis to reduce selection bias due to the retrospective nature of the study, including only patients who underwent Ivor-Lewis esophagectomy with surgical IPD. In this analysis, the BTPD group again demonstrated higher clinical efficacy and was associated with a shorter time to refeeding compared to the IPBT group.

Another important strength of our study is the use of the GOOS as a tool for evaluating the clinical efficacy of endoscopic procedures. The GOOS score is a validated tool for assessing GOO, a syndrome caused by a mechanical obstruction in the gastro-duodenal outflow. To our knowledge, its use in the clinical evaluation of DGCE has not been reported until now. We chose the GOOS score because it is a quick and simple tool that allows us to assess the feeding capacity of patients with a condition like DGCE, which is primarily characterized by the slowed passage of gastric contents into the duodenum.

To the best of our knowledge, no similar comparative studies have been published on this topic, making it difficult to directly compare our results with existing evidence, which has been severely limited by a lack of standardization in DGCE diagnosis, management, and outcomes assessment. Only the study by Bhutani MS et al. evaluated BTPD for DGCE management, reporting a lower CS rate (85%) compared to ours (100%) [16]. We hypothesize that this difference may be due to several factors. First, Bhutani MS and colleagues did not use a standardized protocol for PBD, employing balloons of both 12 mm and 20 mm diameters, whereas in our series, we consistently performed progressive dilation from 18 mm to 20 mm [16]. Additionally, they did not use a standardized protocol for clinical assessment and included in the success group patients who required more than one procedure to achieve symptom relief. Furthermore, in the Bhutani MS et al. series, most patients did not undergo surgical IPD (52%), whereas, in our series, 63.6% of the BTPD group received surgical IPD [16]. Moreover, 67% of patients in the Bhutani MS and colleagues’ series underwent open surgery, which is associated with higher morbidity, while in our study, only 4.5% of cases in the BTPD group involved open surgery.

Although ours is the first comparative study on the endoscopic management of DGCE, it does have some limitations. First, the retrospective and single-center nature of the study limits its generalizability despite being conducted in a tertiary referral center specializing in both luminal digestive endoscopy and esophageal surgery. Another important limitation is that we included all patients affected by DGCE in our comparative study, regardless of the time of onset (early vs. late according to ISDE Classification), which could have influenced the results. To mitigate the impact of the time of onset on clinical outcomes, we included only patients without other surgical complications when performing the Kaplan–Meier analysis with the log–rank test. Additionally, although we used the GOOS score as a tool for clinical evaluation, this score is not currently validated for use in DGCE.

## 5. Conclusions

In conclusion, the endoscopic management of DGCE remains challenging for gastroenterologists for several reasons. In addition to the highly variable factors that contribute to this condition (e.g., anatomical and hormonal), there are no specific guidelines or standardized indications available. As a result, the choice between different treatment options is largely dependent on the experience of the endoscopist and the resources available locally. Results from our retrospective comparative cohort study conducted at a tertiary referral center suggest that the same-session combination of BTPD may provide better and faster symptom relief for patients with both early and late DGCE compared to the individual techniques (IPBT, PBD). More robust data from large, prospective, controlled comparative studies are needed, particularly considering factors such as the type of surgery, IPD, the timing of DGCE onset, and clinical evaluation. The goal should be the standardization of DGCE diagnosis and treatment, always within a multidisciplinary approach.

## Figures and Tables

**Table 1:** Diagnostic Criteria of Delayed gastric conduit emptying (DGCE) [9]. * Early DGCE (E-DGCE): within 14 days of surgery; ** Late DGCE (L-DGCE): later than 14 days after surgery.

Diagnostic Criteria of DGCE [9]			
E-DGCE *	>500 mL diurnal nasogastric tube output measured on the morning of postoperative day five or later (but within 14 days of surgery)	OR	>100% increased gastric tube width on frontal chest X-ray projection (in comparison to baseline chest-X-ray taken on the day of surgery) together with the presence of an air-fluid level
L-DGCE **	The patient should have “quite a bit” or “very much” of at least two of the following symptoms: early satiety/fullness, vomiting, nausea, regurgitation, inability to meet caloric needs by oral intake	AND	Delayed contrast passage on upper GI water-soluble contrast radiogram or on timed barium swallow (until precise evaluation criteria are available, relying on the verdict “delayed contrast passage” by an expert radiologist

**Table 2:** Baseline characteristics of the entire study cohort. BMI, body mass index. ASA score, American Society of Anesthesiologists score. EAC, esophageal adenocarcinoma. SCC, squamous cell carcinoma. CT, chemotherapy. RT, radiotherapy. CRT, Chemo-radiotherapy. ILE, Ivor-Lewis Esophagectomy. MKE, McKeown Esophagectomy. DGCE, Delayed Gastric Conduit Emptying.

Study Cohort (n = 64)	
Sex (male; n, %)	52/64, 81.2%
Age (years; median, IQR)	62 (IQR 55–70)
BMI (kg/m^2^; median, IQR)	26 (IQR 23.1–27.8)
ASA score (n, %)	
I	4/64, 6.2%
II	35/64, 54.7%
III	25/64, 39.1%
Surgical Indication (n, %)	
*Malignant*	62/64, 96.9%
EAC	48/62, 77.4%
SCC	13/62, 21.0%
Leiomyoma	1/62, 1.6%
*Benign*	2/64, 3.1%
Perforation	2/2, 100%
Oncological Staging (n, %)	
Resectable	14/62, 22.6%
Locally Advanced	47/62, 75.8%
Metastatic	1/62, 1.6%
Neoadjuvant treatments (n, %)	
None	15/62, 24.2%
CT	25/62, 40.3%
RT	1/62, 1.6%
CRT	21/62, 33.9%
Type of Surgery (n, %)	
ILE	58/64, 90.6%
MKE	3/64, 4.7%
Total Esophagectomy	3/64, 4.7%
Surgical Approach (n, %)	
Minimally Invasive	56/64, 87.5%
Hybrid	6/64, 9.4%
Open	2/64, 3.1%
DGCE (n, %)	
Early	21/64, 32.81%
Late	43/64, 67.2%

**Table 3:** Comparison of baseline characteristics between the three study groups. IPBT, intra-pyloric botulinum toxin injection. PBD, pneumatic balloon dilation. BTPD, Botulinum Toxin Pneumatic Dilation. BMI, body mass index. ASA score, American Society of Anesthesiologists score. EAC, esophageal adenocarcinoma. SCC, squamous cell carcinoma. CT, chemotherapy. RT, radiotherapy. CRT, Chemo-radiotherapy. ILE, Ivor-Lewis Esophagectomy. MKE, McKeown Esophagectomy. DGCE, Delayed Gastric Conduit Emptying. GOOS, Gastric Outlet Obstruction Score.

Variables	IPBT (n = 18)	PBD (n = 24)	BTPD (n = 22)	*p* Value
Sex (male; n, %)	16/18, 88.9%	19/24, 79.2%	17/22, 77.3%	*p* = 0.61
Age (years; median, IQR)	58 (52–61)	65 (57–72)	65 (60–70)	*p* = 0.08
BMI (kg/m^2^; median, IQR)	26 (22.6–26.7)	25.9 (24.2–28.8)	26.1 (21.1–27.7)	*p* = 0.72
ASA score (n, %)				*p* = 0.16
I	1/18, 5.5%	-	3/22, 13.6%	
II	11/18, 61.1%	16/24, 66.7%	8/22, 36.4%	
III	6/18, 33.3%	8/24, 33.3%	11/22, 50.0%	
Diabetes (n, %)	1/18, 5.6%	3/24, 12.5%	3/22, 13.6%	*p* = 0.68
Surgical indication (n, %)				*p* = 0.53
*Malignant*	17/18, 94.4%	24/24, 100%	21/22, 95.5%	*p* = 0.58
EAC	13/17, 76.5%	19/24, 79.2%	16/21, 76.2%	
SCC	3/17, 17.6%	5/24, 20.8%	5/21, 23.8%	
Leiomyoma	1/17, 5.9%	-	-	
*Benign*	1/18, 5.6%	-	1/22, 4.5%	
Perforation	1/1, 100%	-	1/1, 100%	
Oncological Staging (n, %)				*p* = 0.39
Resectable	6/17, 35.3%	4/24, 16.7%	4/21, 19.1%	
Locally Advanced	11/17, 64.7%	20/24, 83.3%	16/21, 76.2%	
Metastatic	-	-	1/21, 4.7%	
Neoadjuvant treatments (n, %)				*p* = 0.32
None	5/17, 29.4%	6/24, 25%	4/21, 19.1%	
CT	9/17, 52.9%	10/24, 41.7%	6/21, 28.6%	
RT	-	1/24, 4.1%	-	
CRT	3/17, 17.7%	7/24, 29.2%	11/21, 52.3%	
Type of Surgery (n, %)				*p* = 0.26
ILE	17/18, 94.4%	20/24, 83.3%	21/22, 95.5%	
MKE	1/18, 5.6%	1/24, 4.1%	1/22, 4.5%	
Total Esophagectomy	-	3/24, 12.6%	-	
Surgical Approach (n, %)				*p* = 0.79
Minimally Invasive	16/18, 88.9%	20/24, 83.3%	20/22, 91.0%	
Hybrid	2/18, 11.1%	3/24, 12.5%	1/22, 4.5%	
Open	-	1/24, 4.2%	1/22, 4.5%	
Surgical complications (n, %)				*p* = 0.12
None	16/18, 89.0%	16/24, 66.7%	12/22, 54.6%	
Anastomotic Leak	1/18, 5.5%	3/24, 12.5%	8/22, 36.4%	
Conduit necrosis	-	2/24, 8.3%	1/22, 4.5%	
Others	1/18, 5.5%	3/24, 12.5%	1/22, 4.5%	
Pyloric Surgical Interventions (n, %)				*p* = 0.10
None	5/18, 27.8%	17/24, 70.8%	8/22, 36.4%	
Pyloroplasty	-	-	-	
Pyloromyotomy	2/18, 11.1%	2/24, 8.3%	2/22, 9.1%	
Finger fracture	1/18, 5.5%	-	2/22, 9.1%	
Pyloroplasty + Pyloromyotomy	10/18, 55.6%	5/24, 20.9%	10/22, 45.4%	
DGCE (n, %)				*p* < 0.0001
Early	14/18, 77.8%	5/24, 20.8%	2/22, 9.1%	
Late	4/18, 3.2%	19/24, 79.2%	20/22, 90.9%	
GOOS_pre				*p* = 0.33
0	3/18, 16.7%	3/24, 12.5%	3/22, 13.6%	
1	8/18, 44.4%	12/24, 50.0%	5/22, 22.7%	
2	6/18, 33.3%	7/24, 29.2%	8/22, 36.4%	
3	1/18, 5.6%	2/24, 8.3%	6/22, 27.3%	

**Table 4:** Outcomes comparison between the three groups. IPBT, intra-pyloric botulinum toxin injection. PBD, pneumatic balloon dilation. BTPD, Botulinum Toxin Pneumatic Dilation. G-POEM, Gastric-Peroral Endoscopic Myotomy. IQR, inter-quartile range.

Variables	IPBT (n = 18)	PBD (n = 24)	BTPD (n = 22)	*p* Value
Technical Success (n, %)	18/18, 100%	24/24, 100%	22/22, 100%	*p* = 0.65
Clinical Success (n, %)	13/18, 72.2%	22/24, 91.7%	22/22, 100%	***p* = 0.02**
PBD	3/5, 60%	-	-	
BTPD	2/5, 40%	2/2, 100%	-	
Recurrence (n, %)	3/13, 23.1%	5/22, 22.7%	2/22, 9.1%	*p* = 0.41
IPBT	-	1/5, 20%	-	
PBD	-	2/5, 40%	-	
BTPD	-	2/5, 40%	-	
G-POEM	3/3, 100%	-	1/2, 50%	
Pyloromyotomy+	-	-	1/2, 50%	
pyloroplasty				
Follow up	374 (208–739)	184 (35–710)	230 (144–589)	*p* = 0.19
(days; median/IQR)				

**Table 5:** Comparison of baseline characteristics for subgroup analysis. IPBT, intra-pyloric botulinum toxin injection. BTPD, Botulinum Toxin Pneumatic Dilation. BMI, body mass index. ASA score, American Society of Anesthesiologists score. EAC, esophageal adenocarcinoma. SCC, squamous cell carcinoma. CT, chemotherapy. RT, radiotherapy. CRT, Chemo-radiotherapy. DGCE, Delayed Gastric Conduit Emptying. GOOS, Gastric Outlet Obstruction Score.

Variables	IPBT (n = 12)	BTPD (n = 14)	*p* Value
Sex (male; n, %)	12/12, 100%	11/14, 78.6%	*p* = 1
Age (years; median, IQR)	58 (48–64)	65 (59–71)	*p* = 0.06
BMI (kg/m^2^; median, IQR)	26.2 (22.0–28.9)	26.1 (21.8–28.1)	*p* = 0.24
ASA score (n, %)			*p* = 0.57
I	1/12, 8.3%	3/14, 21.5%	
II	6/12, 50.0%	5/14, 35.7%	
III	5/12, 41.7%	6/14, 42.8%	
Diabetes (n, %)	1/12, 8.3%	2/14, 13.6%	*p* = 0.64
Surgical indication (n, %)			*p* = 0.35
*Malignant*	12/12, 100%	13/14, 92.8%	*p* = 0.38
EAC	10/12, 83.4%	10/13, 76.9%	
SCC	1/12, 8.3%	3/13, 23.1%	
Leiomyoma	1/12, 8.3%	-	
*Benign*	-	1/14, 7.2%	
Perforation	-	1/1, 100%	
Oncological Staging (n, %)			*p* = 0.56
Resectable	4/12, 33.4%	3/13, 23.1%	
Locally Advanced	8/12, 66.6%	9/13, 69.2%	
Metastatic	-	1/13, 7.7%	
Neoadjuvant treatments (n, %)			*p* = 0.331
None	3/12, 25.0%	4/13, 28.6%	
CT	7/12, 58.3%	4/13, 28.6%	
RT	-	-	
CRT	2/12, 16.7%	5/13, 35.8%	
Surgical Approach (n, %)			*p* = 0.64
Minimally Invasive	11/12, 91.7%	12/14, 85.8%	
Hybrid	1/12, 8.3%	1/14, 7.1%	
Open	-	1/14, 7.1%	
Surgical complications (n, %)			*p* = 0.15
None	11/12, 91.7%	8/14, 51.2%	
Anastomotic Leak	-	4/14, 28.6%	
Conduit necrosis	-	1/14, 7.1%	
Others	1/12, 8.3%	1/14, 7.1%	
Pyloric Surgical Interventions (n, %)			*p* = 0.89
Pyloromyotomy	2/12, 13.3%	2/14, 10.5%	
Finger fracture	1/12, 8.3%	2/14, 10.5%	
Pyloroplasty + Pyloromyotomy	9/12, 26.7%	10/14, 42.1%	
DGCE (n, %)			
Early	7/12, 58.3%	3/14, 21.4%	*p* = 0.054
Late	5/12, 41.7%	11/14, 78.6%	
GOOS_pre			
0	2/12, 16.7%	2/14, 14.3%	*p* = 0.39
1	6/12, 50.0%	3/14, 21.4%	
2	4/12, 33.3%	5/14, 35.7%	
3		4/14, 28.6%	

**Table 6:** Outcomes comparison of the subgroup analysis. IPBT, intra-pyloric botulinum toxin injection. PBD, pneumatic balloon dilation. BTPD, Botulinum Toxin Pneumatic Dilation. G-POEM, Gastric-Peroral Endoscopic Myotomy. IQR, inter-quartile range.

Variables	IPBT (n = 12)	BTPD (n = 14)	*p* Value
Technical Success (n, %)	12/12, 100%	14/14, 100%	*p* = 0.65
Clinical Success (n, %)	9/12, 72.2%	14/14, 100%	***p* = 0.04**
PBD	1/3, 33.3%%	-	
BTPD	2/3, 66.7%	-	
Recurrence (n, %)	3/9, 33.3%	1/14, 7.1%	*p* = 0.11
BTPD	1/3, 33.3%	-	
G-POEM	1/3, 33.3%	-	
Pyloromyotomy+	1/3, 33.3%	1/1, 100%	
pyloroplasty			
Follow up	313 (208–608)	197 (65–499)	*p* = 0.12
(days; median/IQR)			

## Data Availability

The original contributions presented in the study are included in the article, further inquiries can be directed to the corresponding author.

## References

[1] Sheikh M., Roshandel G., McCormack V., Malekzadeh R. (2023). Current Status and Future Prospects for Esophageal Cancer. Cancers.

[2] van der Wilk B.J., Hagens E.R.C., Eyck B.M., Gisbertz S.S., van Hillegersberg R., Nafteux P., Schröder W., Nilsson M., Wijnhoven B.P.L., Lagarde S.M. (2022). Outcomes after totally minimally invasive versus hybrid and open Ivor Lewis oesophagectomy: Results from the International Esodata Study Group. Br. J. Surg..

[3] Low D.E., Alderson D., Cecconello L., Chang A.C., Darling G.E., D’Journo X.B., Griffin S.M., Hölscher A.H., Hofstetter W.L., Jobe B.A. (2015). International Consensus on Standardization of Data Collection for Complications Associated with Esophagectomy. Ann. Surg..

[4] Yang H.C., Choi J.H., Kim M.S., Lee J.M. (2020). Delayed Gastric Emptying after Esophagectomy: Management and Prevention. Korean J. Thorac. Cardiovasc. Surg..

[5] Pattynama L.M.D., Eshuis W.J., Seewald S., Pouw R.E. (2024). Multi-modality management of defects in the gastrointestinal tract: Where the endoscope meets the scalpel: Endoscopic vacuum therapy in the upper gastrointestinal tract. Best Pract. Res. Clin. Gastroenterol..

[6] Dell’Anna G., Fanti L., Fanizza J., Barà R., Barchi A., Fasulo E., Elmore U., Rosati R., Annese V., Laterza L. (2024). VAC-Stent in the Treatment of Post-Esophagectomy Anastomotic Leaks: A New “Kid on the Block” Who Marries the Best of Old Techniques—A Review. J. Clin. Med..

[7] Tham J.C., Pournaras D.J., Alcocer B., Forbes R., Ariyarathenam A.V., Humphreys M.L., Berrisford R.G., Wheatley T.J., Chan D., Sanders G. (2022). Gut hormones profile after an Ivor Lewis gastro-esophagectomy and its relationship to delayed gastric emptying. Dis. Esophagus.

[8] Mandarino F.V., Sinagra E., Raimondo D., Danese S. (2023). The Role of Microbiota in Upper and Lower Gastrointestinal Functional Disorders. Microorganisms.

[9] Konradsson M., van Berge Henegouwen M.I., Bruns C., Chaudry M.A., Cheong E., Cuesta M.A., Darling G.E., Gisbertz S.S., Griffin S.M., Gutschow C.A. (2020). Diagnostic criteria and symptom grading for delayed gastric conduit emptying after esophagectomy for cancer: International expert consensus based on a modified Delphi process. Dis. Esophagus.

[10] Klevebro F., Konradsson M., Han S., Luttikhold J., Nilsson M., Lindblad M., Andersson M. (2023). ERAS guidelines-driven upper gastrointestinal contrast study after esophagectomy can detect delayed gastric conduit emptying and improve outcomes. Surg. Endosc..

[11] Zhang R., Zhang L. (2019). Management of delayed gastric conduit emptying after esophagectomy. J. Thorac. Dis..

[12] Okada N., Kinoshita Y., Nishihara S., Kurotaki T., Sato A., Kimura K., Kushiya H., Umemoto K., Furukawa S., Yamabuki T. (2023). PYloroplasty versus No Intervention in GAstric REmnant REconstruction after Oesophagectomy: Study protocol for the PYNI-GAREREO phase III randomized controlled trial. Trials.

[13] Eldaif S.M., Lee R., Adams K.N., Kilgo P.D., Gruszynski M.A., Force S.D., Pickens A., Fernandez F.G., Luu T.D., Miller D.L. (2014). Intrapyloric Botulinum Injection Increases Postoperative Esophagectomy Complications. Ann. Thorac. Surg..

[14] Bagheri R., Fattahi S.H., Haghi S.Z., Aryana K., Aryanniya A., Akhlaghi S., Riyabi F.N., Sheibani S. (2013). Botulinum toxin for prevention of delayed gastric emptying after esophagectomy. Asian Cardiovasc. Thorac. Ann..

[15] Mertens A., Gooszen J., Fockens P., Voermans R., Gisbertz S., Bredenoord A., van Berge Henegouwen M.I. (2021). Treating Early Delayed Gastric Tube Emptying after Esophagectomy with Pneumatic Pyloric Dilation. Dig. Surg..

[16] Bhutani M.S., Ejaz S., Cazacu I.M., Singh B.S., Shafi M., Stroehlein J.R., Mehran R.J., Walsh G., Vaporciyan A., Swisher S.G. (2022). Endoscopic Intrapyloric Botulinum Toxin Injection with Pyloric Balloon Dilation for Symptoms of Delayed Gastric Emptying after Distal Esophagectomy for Esophageal Cancer: A 10-Year Experience. Cancers.

[17] Vanella G., Dell’Anna G., Capurso G., Maisonneuve P., Bronswijk M., Crippa S., Tamburrino D., Macchini M., Orsi G., Casadei-Gardini A. (2023). EUS-guided gastroenterostomy for management of malignant gastric outlet obstruction: A prospective cohort study with matched comparison with enteral stenting. Gastrointest Endosc..

[18] Fugazza A., Andreozzi M., Aghdaei H.A., Insausti A., Spadaccini M., Colombo M., Carrara S., Terrin M., De Marco A., Franchellucci G. (2024). Management of Malignant Gastric Outlet Obstruction: A Comprehensive Review on the Old, the Classic and the Innovative Approaches. Medicina.

[19] Nass K.J., Zwager L.W., van der Vlugt M., Dekker E., Bossuyt P.M.M., Ravindran S., Thomas-Gibson S., Fockens P. (2022). Novel classification for adverse events in GI endoscopy: The AGREE classification. Gastrointest Endosc..

[20] Mandarino F.V., Testoni S.G.G., Barchi A., Azzolini F., Sinagra E., Pepe G., Chiti A., Danese S. (2023). Imaging in Gastroparesis: Exploring Innovative Diagnostic Approaches, Symptoms, and Treatment. Life.

[21] Gaur P., Swanson S.J. (2014). Should we continue to drain the pylorus in patients undergoing an esophagectomy?. Dis. Esophagus.

[22] Hajibandeh S., Hajibandeh S., McKenna M., Jones W., Healy P., Witherspoon J., Blackshaw G., Lewis W., Foliaki A., Abdelrahman T. (2023). Effect of intraoperative botulinum toxin injection on delayed gastric emptying and need for endoscopic pyloric intervention following esophagectomy: A systematic review, meta-analysis, and meta-regression analysis. Dis. Esophagus.

[23] Arya S., Markar S.R., Karthikesalingam A., Hanna G.B. (2015). The impact of pyloric drainage on clinical outcome following esophagectomy: A systematic review. Dis. Esophagus.

[24] Loo J.H., Ng A.D.R., Chan K.S., Oo A.M. (2023). Outcomes of Intraoperative Pyloric Drainage on Delayed Gastric Emptying Following Esophagectomy: A Systematic Review and Meta-analysis. J. Gastrointest Surg..

[25] Abdelrahman M., Ariyarathenam A., Berrisford R., Humphreys L., Sanders G., Wheatley T., Chan D.S.Y. (2022). Systematic review and meta-analysis of the influence of prophylactic pyloric balloon dilatation in the prevention of early delayed gastric emptying after oesophagectomy. Dis. Esophagus.

[26] Mandarino F.V., Sinagra E., Barchi A., Danese S. (2024). The Triple-S Advantage of Endoscopic Management in Gastrointestinal Surgery Complications: Safe, Successful, and Savings-Driven. Life.

[27] Debourdeau A., Vitton V., Gonzalez S., Collet H., Al Tabaa Y., Barthet M., Gonzalez J.M. (2024). Prognostic value of preoperative intragastric meal distribution in gastric emptying scintigraphy for long-term success of gastric peroral endoscopic myotomy in gastroparesis. Gastrointest. Endosc..

[28] Aziz M., Gangwani M.K., Haghbin H., Dahiya D.S., Sohail A.H., Kamal F., Lee-Smith W., Adler D.G. (2023). Gastric peroral endoscopic myotomy versus surgical pyloromyotomy/pyloroplasty for refractory gastroparesis: Systematic review and meta-analysis. Endosc. Int. Open.

[29] Chan F.S.Y., Wong I.Y.H., Chan D.K.K., Wong C.L.Y., Law B.T.T., Chow V.L.Y., Law S. (2022). Gastric peroral endoscopic myotomy for delayed gastric conduit emptying after pharyngo-laryngo-esophagectomy: A case report. Hong Kong Med. J..

[30] Malik S., Loganathan P., Khan K., Mohan B.P., Adler D.G. (2024). Efficacy and Safety of Gastric Peroral Endoscopic Myotomy across Different Etiologies of Gastroparesis: A Systematic Review and Meta-Analysis. Gastrointest. Endosc..

